# The Genetic Structure and Diversity of Different Pigeon Breeds Based on a 5 K Single Nucleotide Polymorphism Chip

**DOI:** 10.3390/ani15192864

**Published:** 2025-09-30

**Authors:** Haobin Hou, Xin Li, Xiaoliang Wang, Xia Cai, Yingying Tu, Wenwei Lv, Xiaohui Shen, Changsuo Yang, Junfeng Yao

**Affiliations:** 1 Institute of Animal Science and Veterinary Medicine, Shanghai Academy of Agricultural Sciences, Shanghai 201106, China; houhaobean@163.com (H.H.);; 2 National Poultry Engineer Research Center, Shanghai 201106, China

**Keywords:** pigeon, liquid chip, genetic structure, genetic diversity

## Abstract

**Simple Summary:**

This study developed a single-nucleotide polymorphism chip to analyze genomic relationships and genetic diversity among 10 pigeon breeds (meat, racing, fancy). Principal component analysis clustered them into three groups: homing and Tarim pigeons; Dianzi and Xinjiang Roller varieties; and commercial meat breeds (Euro-pigeon, Danish King, Silver King, etc.). Phylogenetic trees showed close ties between homing/Tarim and Dianzi/Xinjiang Roller, while Yellow and Red Carneau had similar genetic backgrounds despite feather color differences. Danish King and Silver King, though similar in plumage, were genetically distinct. The chip effectively differentiates pigeon populations, aiding germplasm resource identification, especially pure breed verification and new resource exploration.

**Abstract:**

China has the largest population of pigeons globally, particularly for commercial meat production. Due to insufficient emphasis on bloodline preservation, there is a significant occurrence of breed hybridization, which presents challenges to the differentiation and identification of various pigeon breeds. In this study, a single-nucleotide polymorphism chip was developed to elucidate genomic relationships and genetic diversity among 10 pigeon breeds, encompassing meat, racing, and ornamental varieties. Principal component analysis revealed that this resource population could be classified into three major clusters: homing and Tarim pigeons; the Dianzi (DZ) and Xinjiang Roller (XR) varieties; and commercial meat pigeon breeds, including the Euro-pigeon (EP), Danish King (DK), Silver King (SK), Yellow Carneau (YC), Red Carneau (RC), and Taishen (TS) varieties. Phylogenetic tree analysis indicated that the HP, TR, DZ, and XR varieties clustered into a large group. Of these, the HP and TR groups and the DZ and XR group were closely genetically related. Other meat pigeon varieties clustered into a large group. The genetic relationship between the YC and RC pigeons was intertwined, suggesting that although there were differences in feather color, the genetic backgrounds are similar. The phylogenetic tree results also demonstrated that the DK and SK pigeons had a considerable genetic distance, indicating that although the feather color was similar, the birds belong to two distinct genetic groups. The Pigeon 5 K liquid chip can effectively discriminate among different pigeon populations and provides a method for the identification and evaluation of pigeon germplasm resources, especially for pure breed identification and exploration of new resources.

## 1. Introduction

Artificial selection has given rise to many varieties of domesticated pigeons (fancy, carrier, and squabs) with unique phenotypes. Although different varieties of pigeons can be easily distinguished based on appearance and body size, the genetic background, relationships, and diversity among breeds are complex, rendering identification especially difficult. As compared to other livestock and poultry, there is no strict phylogenetic basis for the classification of pigeon breeds, and there is often no record of the specific birds crossed for the development of new varieties [1]. The breeding quantity of meat pigeons in China ranks first in the world, owing to the abundant genetic resources, mostly dominated by imported varieties. After these varieties entered China, driven by the consumer market and breeding environment, a series of new types have emerged. At the same time, there has been extensive hybridization, resulting in mixed bloodlines of some varieties. With the advancement of genomic sequencing and bioinformatics technologies, the parsing of genomic information can disclose the evolutionary relationships among various pigeon breeds and genetic variances among individuals, thereby providing a theoretical foundation for the management of bloodlines and kinship as well as conservation efforts. However, pigeons, even those for commercial meat production, tend to be monogamous. Theoretically, as compared to other livestock and poultry, pairing one male with multiple females has certain advantages in genetic diversity and reduces inbreeding.

Although molecular genetics and biotechnology have been successfully applied for the identification of livestock and poultry, research on domestic pigeons is relatively limited. The cytochrome oxidase subunit I (COI) gene is recognized as a reliable marker for the identification of members of the Columbidae family of pigeons and doves [2]. The phylogenetic relationships and genetic diversity among different pigeon breeds have been recently investigated through mitochondrial DNA sequence analysis of the COI gene [3]. With the development of whole-genome sequencing (WGS), the first pigeon reference genome was assembled [4]. Genetic analysis of 70 pigeon breeds revealed convergent evolution of derived traits and elucidated the geographic origins of breeds in India and the Middle East, demonstrating that racing pigeon breeds have significantly contributed to wild pigeon populations [5]. Simplified genotyping-by-sequencing (GBS) is an efficient method to analyze the genetic structure and diversity of pigeons [1]. WGS has unveiled the genetic relationships, structure, and diversity among the major pigeon genetic resource groups in China [6]. These advancements in pigeon genomics have further deepened understanding of the selection and domestication of this model species, providing a theoretical foundation for the protection and utilization of pigeon genetic resources.

The development of high-throughput sequencing technology has provided a wealth of genomic information on livestock and poultry species. Although the high cost has hindered commercial application, resequencing, simplified WGS, and bioinformatics analysis have been applied to elucidate the genetic diversity and relationships among and within breeds. Genotyping by target sequencing (GBTS) liquid chips, as an economical and efficient alternative to WGS and GBS, are widely used for analysis of various livestock species [7]. Based on high-throughput sequencing technology, GBTS is a liquid phase chip technology that has been applied for resource identification, kinship analysis, molecular marker-assisted breeding, and whole-genome selection of livestock and poultry. As compared to endonuclease-mediated GBS technology, GBTS has stronger targeting and greater site coverage. Liquid phase chips have improved the efficiency and accuracy of screening of molecular markers for the identification of pigeon species and molecular marker-assisted breeding.

## 2. Materials and Methods

### 2.1. Ethical Approval

The protocol of the animal study was approved by the Institutional Animal Care and Use Committee of Shanghai Academy of Agricultural Sciences (approval no. SAAS-SL-2022014) and conducted in accordance with the Guide for the Care and Use of Laboratory Animals.

### 2.2. Screening of the Pigeon 5 K Genetic Markers

Based on the Variant Call Format files of pigeon WGS data and the sequencing data of simplified genomes, screening of population single-nucleotide polymorphisms (SNPs) was conducted to obtain SNP loci for analysis. All SNPs with a quality value of 20, indicating a sequencing error rate greater than 1%, were filtered out. The SNP loci were required to be separated by at least 5 bp, and the coverage depth of SNPs was within the range of [1/3, 5] times the average depth. Insertion/deletion polymorphisms were removed, while SNPs with two alleles, coverage depth ≥ 5×, minor allele frequency (MAF) ≥ 0.05, and missing rate of <0.25 were retained. SNP loci with a heterozygosity rate < 0.5 were also filtered out. Then, SNP loci were evaluated using the following parameters: length, 110 bp; GC content, 30–70%; number of homologous regions, 5; dimensions, 2; size, 120 bp; and distance, 10 bp. SNP loci were sorted and screened based on a high MAF, low missing rate, and low heterozygosity rate to obtain background loci for testing. Based on the liquid-phase probe capture technology, GenoBaits, a 5 K locus panel was designed and synthesized for testing. From the test results, 5 K loci that met the requirements were selected, and a final usable pigeon panel was synthesized for use with a pigeon 5 K marker detection kit.

### 2.3. Design of Liquid-Phase Probes

**Figure 1:**
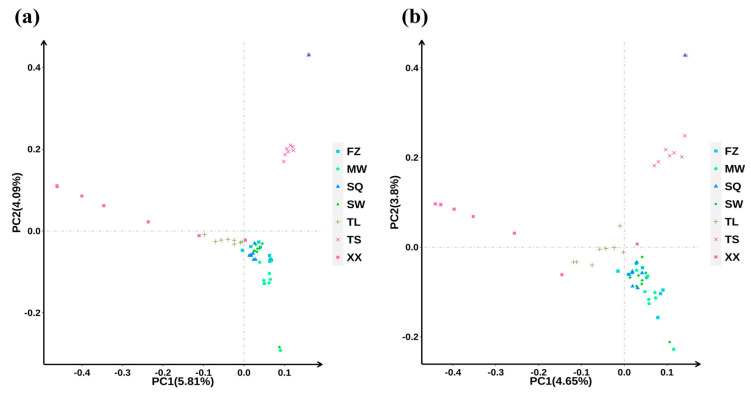
Evaluation of consistency in PCA between whole-genome resequencing data and the 5 K liquid-phase chip for pigeons. (**a**) PCA was conducted based on the Variant Call Format files derived from whole-genome resequencing data. (**b**) PCA was performed using data obtained from the 5 K liquid-phase chip specific for pigeons.

The length of the probe was 110 bp, the GC content was controlled within the range of 30% to 70%, and the number of homologous regions could not exceed 5. For the selected SNP loci, two nucleotide sequences with 60% to 70% overlap were designed. Finally, 54,845,483 loci were selected as the final locus set for the pigeon 5 K liquid-phase chip. Since assembly was not conducted at the chromosome level, the pigeon reference genome was integrated into six sequence sets to facilitate analysis (Figure 1).

### 2.4. Targeted Sequencing and Genotyping of Pigeon DNA Samples

Blood samples were collected from the wing veins of 137 pigeons belonging to the 10 following groups: Danish King [8], *n* = 18; Dianzi (DZ), *n* = 14; Euro-pigeon (EP), *n* = 11; Homing Pigeon (HP), *n* = 9; Red Carneau (RC), *n* = 18; Silver King (SK), *n* = 18; Tarim Pigeon (TR), *n* = 14; Taisen (TS), *n* = 14; Xinjiang Roller (XR), *n* = 4; and Yellow Carneau (YC), *n* = 17. These pigeon breeds were from Shanghai Jinhuang Pigeon Industry Co., Ltd. DNA was extracted, and targeted sequencing libraries were constructed. After library construction, the initial quantification was performed using a Qubit 2.0 fluorometer (Thermo Fisher Scientific, Waltham, MA, USA), and the effective concentration of the libraries was determined by quantitative polymerase chain reaction to ensure quality prior to sequencing.

### 2.5. Basic Statistics of Genetic Diversity

Basic statistics of genetic diversity parameters were conducted for all SNP markers, including MAF, polymorphism information content (PIC), observed number of alleles (Ao), expected number of alleles (Ae), observed heterozygosity (Ho), and expected heterozygosity (He).

### 2.6. Population Analysis

Population analysis encompassed five components: principal component analysis (PCA), population structure analysis, construction of evolutionary trees, linkage disequilibrium [5] decay analysis, and kinship analysis. PCA based on the filtered SNP markers was conducted to obtain the variance explanation rate and the score matrix of each principal component (PC) using Genome-wide Complex Trait Analysis software (v1.92.4; https://yanglab.westlake.edu.cn/software/gcta/, accessed on 21 June 2024). The key information extracted from the SNP data was classified from PC1 to PC3 in descending order of effect size. The results are presented as pairwise scatter plots of the first three PCs. Admixture software (v1.3; https://dalexander.github.io/admixture/, accessed on 21 June 2024) was utilized to infer the population structure, with the assumed number of clusters (K value) of samples ranging from 1 to 15, followed by clustering. The optimal number of clusters was determined based on the cross-validation (CV) error, with the K value corresponding to the smallest CV error representing the optimal number of clusters. An evolutionary tree was constructed using the neighbor-joining method. Based on the filtered SNP markers, the LD size (r^2^) between each pair of markers was calculated using PopLDdecay 3.42 software (https://github.com/BGI-shenzhen/PopLDdecay/, accessed on 11 July 2024) and plotted as the variation with increasing distance. Based on the screened SNP markers, an evolutionary tree was constructed using the neighbor-joining method with Molecular Evolutionary Genetics Analysis-X software (model, p-distance; bootstrap value, 1000×; https://www.megasoftware.net/, accessed on 11 July 2024).

## 3. Results

**Figure 2:**
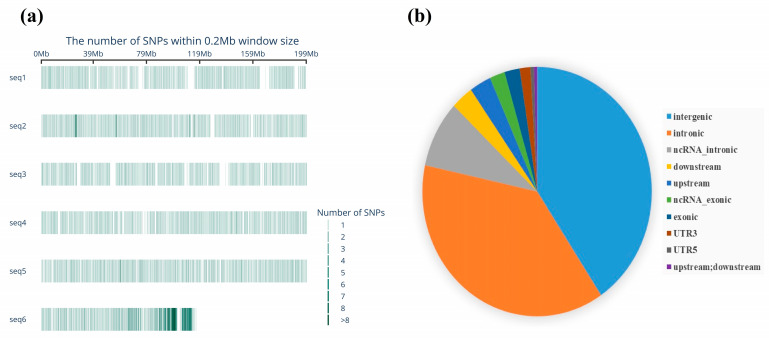
Development of a 5 K marker detection kit for pigeons. (**a**) Chromosomal distribution map of SNP markers for the pigeon 5 K genotyping array. (**b**) The distribution of SNP markers across gene structures.

The site selection and evaluation process identified 6140 sites for analysis. Subsequently, based on capture stability, 5484 SNP sites and two insertion/deletion sites were finally selected for development of the pigeon 5 K marker chip. PCA of the whole-genome resequencing data and 5 K chip data from 55 pigeons demonstrated a high degree of concordance in population stratification, indicating that the loci selected for the 5 K chip exhibited satisfactory genome-wide representativeness (Figure 1a,b). The SNP markers were uniformly distributed across six sequences (Figure 2a), with the majority located within intronic and intergenic regions of genes (Figure 2b). The average site detection rate of the pigeon 5 K marker chip was 99.913%. The test results met the requirements for the development of the pigeon 5 K marker detection kit.

### 3.1. Basic Statistics of Genetic Diversity

**Figure 3:**
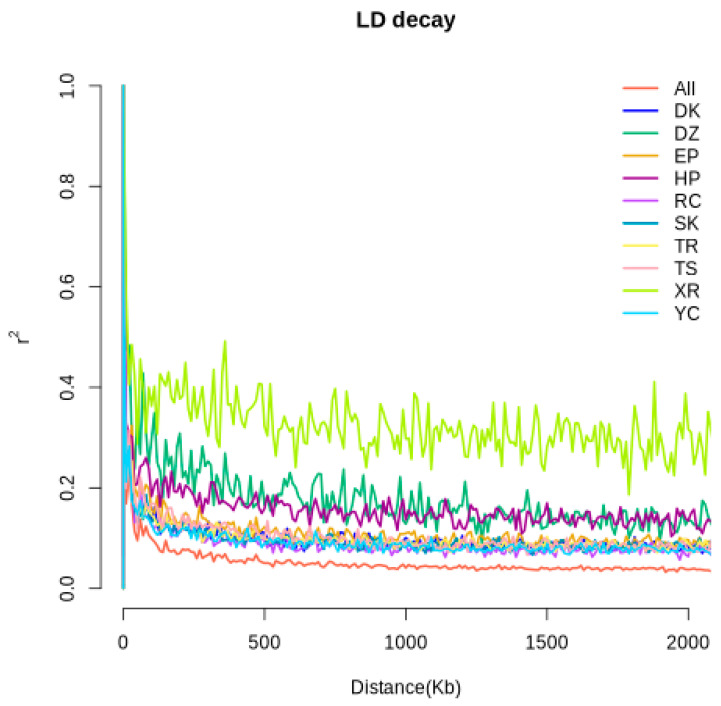
LD decay plots across different pigeon breeds.

The genetic diversity indices are presented in Table 1. The MAF, PIC, Ao, Ae, Ho, and He values of the DZ and XR groups were relatively low. The PIC values in terms of numerical magnitude were SK > TR > RC > YC > DK > TS > EP > HP > XR > DZ (Table 1). LD analysis of the genomes of the 10 pigeon populations was conducted using the 5 K chip to explore the relationship between the LD coefficient at different genomic loci and the marker spacing (Figure 3). The results demonstrated that distinct populations displayed obvious differences in LD. The XR group had the highest attenuation rate and the lowest genetic diversity, followed by the DZ and HP groups, which had relatively high attenuation rates and relatively low genetic diversity. The LD attenuation rates of the seven pigeon breeds DK, EP, RC, SK, TR, TS, and YC were relatively similar, and genetic diversity was relatively high.

### 3.2. PCA

**Figure 4:**
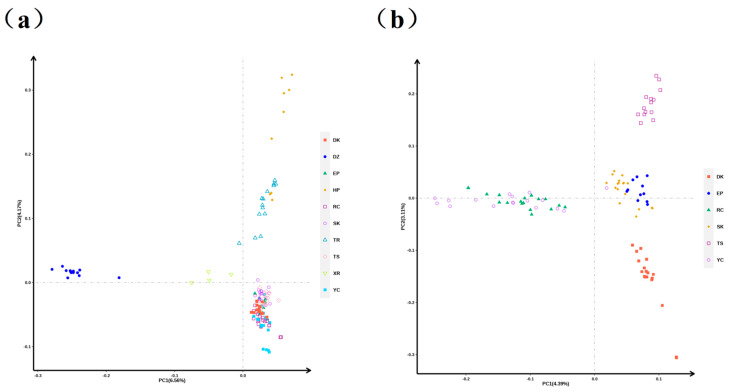
PCA of different pigeon breeds. (**a**) PCA of 10 pigeon populations. (**b**) PCA of commercial meat pigeon populations.

Significant differences in genetic variation were observed among the 10 pigeon populations. PC1 accounted for 6.56% of the genetic variation among the populations, while PC2 accounted for 4.17% (Figure 4a). The results indicated the formation of five groups: DZ, XR, TR, HP, and commercial meat pigeons. Among these, a few TR and HP individuals and the commercial meat pigeon varieties had close genetic relationships. PCA of the meat pigeon populations using the 5 K chip found that PC1 and PC2 explained 4.39% and 3.11% of the genetic variation among the populations, respectively (Figure 4b). The RC and YC groups had a close genetic relationship, while the TS, EP, SK, and DK groups formed four independent groups.

### 3.3. Population Structure Analysis

**Figure 5:**
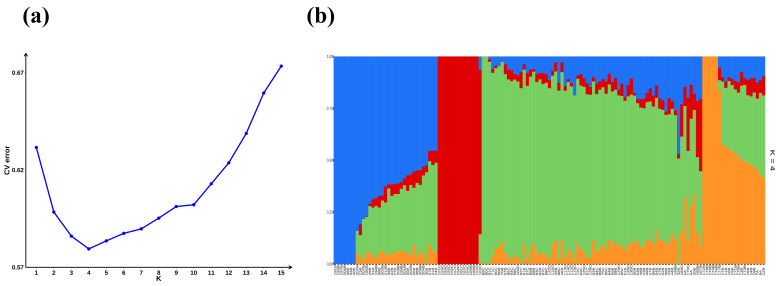
Analysis of population structure. (**a**) Line chart depicting the CV error rate. (**b**) Bar chart illustrating the genetic composition of samples.

To further analyze the population structure of the studied groups, Admixture software with K values of 1–15 was used to assess the population admixture. According to the cross-validation error, as the K value increased, the CV error first decreased and then increased. The rate of decrease was relatively fast at K values of 1–4, whereas the rate of increase was relatively fast at K values of 10–15. The CV error was relatively stable at K values of 4–10. K = 4 was the optimal number of clusters (Figure 5a). The main blue, red, green, and yellow genetic clusters were the Carneau, DZ, King, and Racing pigeons, respectively (Figure 5b).

### 3.4. Phylogenetic Tree Construction

**Figure 6:**
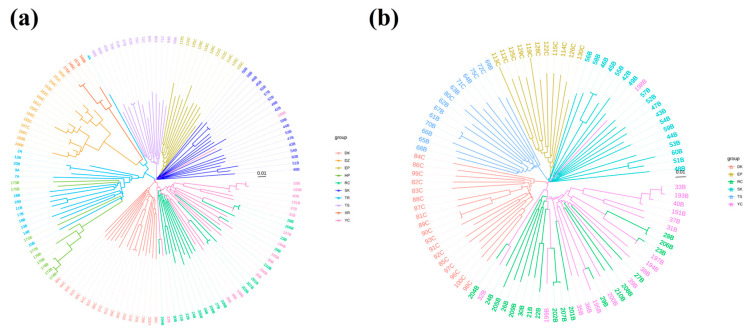
Construction of a phylogenetic tree. (**a**) Construction and analysis of a phylogenetic tree of 10 pigeon groups. (**b**) Construction and analysis of a phylogenetic tree for commercial meat pigeon populations.

Phylogenetic tree analysis shows that the 10 pigeon breeds can be divided into seven groups (Figure 6a), which included four independent groups formed by the DK, SK, EP, and TS groups, while the DZ and XR, HP and TR, and RC and YC groups formed independent clusters. Phylogenetic analysis of the commercial meat pigeon populations revealed that the DK, RC, and WC groups formed a cluster, whereas the TS, EP, and SK groups formed a distinct clade (Figure 6b).

## 4. Discussion

Previous studies have explored the applicability of chicken microarray chips for genetic analysis of pigeon breeds. Although the use of chicken microarray chips for analysis of pigeon samples is technically feasible [9], the detection rate was only 47.4% due to significant interspecies differences. Therefore, it is imperative to develop a specialized SNP chip tailored for pigeons. In this study, a 5 K SNP chip was successfully developed for the identification and evaluation of pigeon genetic resources. This chip enabled us to assess the genetic diversity of ornamental, racing, and commercial meat pigeon varieties in addition to local breeds. Analysis of mitochondrial DNA revealed relatively low levels of genetic diversity among ornamental pigeons [10]. A reduced effective population size of animal species poses a significant risk to long-term viability, especially for those with a high genetic load [11]. The domestic pigeon exhibits a pattern consistent with the primary trends observed in its lineage, indicating that the reduced genetic diversity may be attributable to recurrent population bottlenecks [1]. The present study included the ornamental pigeon breeds DZ and XR. Notably, the DZ breed is a traditional Chinese ornamental pigeon with a documented breeding history spanning several centuries [12]. The XR breed is a variety of Fantail pigeon originating from the Xinjiang region of China. Due to the small population size and intense artificial selection, both the DZ and XR breeds exhibit relatively low genetic diversity, which has led to lower vitality and environmental adaptability. Therefore, for conservation of these pigeon breed resources, it is imperative to maintain a sufficiently large population size to preserve genetic diversity. Analysis of the genetic diversity of Polish racing pigeons using microsatellite markers revealed a relatively satisfactory level of diversity (Ho = 0.623, He = 0.684) [8]. In the present study, genetic diversity was significantly higher for racing pigeons than ornamental pigeons (Ho = 0.326, He = 0.301). LD analysis indicated that the decay rate was marginally lower for the HP group than the DZ group. Commercial breeding of meat pigeons in China began relatively recently, with most breeds (King, Carneau, and Mirthys) introduced from the United States and Europe. Analysis of the genetic diversity of mitochondrial DNA revealed that the Mediterranean pigeon groups (Carneau, Mirthys, Mondain, and Runt) had the highest haplotype diversity, while meat pigeon breeds from the United States and Hungary had comparatively lower genetic diversity [3]. The genetic diversity of commercial pigeon breeds examined in this study was higher than the ornamental and racing pigeon breeds. Specifically, SK, Carneau, and DK pigeons had notably high levels of genetic diversity, indicating substantial potential for breeding selection. The TR breed, a unique local germplasm resource from Xinjiang, China, was domesticated from wild pigeons and has a long-standing history of cultivation [13]. The findings of this study demonstrate that the TR breed had a relatively high level of genetic diversity, which provides a robust genetic foundation for adaptation to the local environment and is conducive to the conservation and sustainable development of local pigeon breeds.

In contrast to other domesticated animals, the evolutionary history of pigeons is replete with hybridization [1]. Given the complex genetic background, it is scientifically inadequate to differentiate the relationships among pigeon breeds based solely on morphological and phenotypic characteristics. PCA of the 5 K chip revealed four major clusters: ornamental, racing, and commercial meat pigeon breeds, in addition to local breeds. These groups exhibit distinct functional purposes—ornamental display, racing performance, and meat production—leading to significant genetic differentiation. When conducting PCA of a large sample of commercial meat pigeons separately, different genetic types were discerned. However, the RC and YC populations remained indistinguishable. Phylogenetic tree analysis yielded consistent results. Notably, the Carneau pigeon was originally bred in France for meat production, with primary feather color variants including white, red, and yellow. Previous studies of feather color dilution have indicated that the *SLC45A2* gene mutation can change the plumage from red to yellow [14].

Despite the markedly different feather colors of RC and YC pigeons, their genetic backgrounds are highly intermingled, indicating that these two breeds share a similar genetic makeup except for the genes responsible for feather color. In contrast, DK and SK pigeons, both characterized by silver plumage, exhibit a considerable genetic distance as revealed by PCA and phylogenetic tree analyses. The feather color of pigeons is determined by a few genes, whereas body morphology, appearance traits, and production characteristics are complex polygenic traits. In this study, DK pigeons were larger with a more upturned tail, resembling exhibition King pigeons, while Silver King pigeons were slightly smaller with superior reproductive performance. TS, a self-distinguishing breed, was more closely related to the EP and SK breeds. Roller pigeons are renowned for their distinctive aerial rolls during flight [15]. Phylogenetic analysis indicated that XR pigeons are closely related to DZ pigeons but have undergone significant differentiation, clustering together with the HP and TR varieties into one major group. It is hypothesized that these two ornamental pigeon breeds may have been domesticated from local pigeon varieties. Additionally, the HP and TR varieties exhibited a relatively close genetic distance and share common ancestral lineages, providing valuable genomic data to clarify hybridization and differentiation among pigeon breeds.

## 5. Conclusions

We developed a 5 K liquid-phase genotyping chip to evaluate pigeon germplasm resources and elucidate complex genetic backgrounds. Analysis of the genetic diversity and population structure of 10 distinct pigeon groups with this chip revealed the genetic diversity and differentiation characteristics of pigeon populations with diverse functional uses. These findings offer a robust theoretical foundation for the conservation, evaluation, and sustainable utilization of pigeon germplasm resources.

## Figures and Tables

**Table 1:** Comprehensive statistical analysis of genetic diversity parameters across diverse pigeon breeds.

Group	MAF	PIC	Ao	Ae	Ho	He
All	0.2574	0.2965	2.0034	1.6084	0.3448	0.3676
DK	0.2434	0.2718	1.9854	1.5624	0.3622	0.3368
DZ	0.1605	0.1724	1.6246	1.3672	0.2494	0.2152
EP	0.2421	0.2653	1.9568	1.5558	0.3354	0.3297
HP	0.2251	0.2418	1.8834	1.5129	0.3256	0.3014
RC	0.2522	0.2771	1.9886	1.5816	0.3534	0.3448
SK	0.2607	0.2870	1.9943	1.6039	0.3794	0.3576
TR	0.2600	0.2808	1.9819	1.5974	0.3677	0.3507
TS	0.2422	0.2672	1.9701	1.5575	0.3498	0.3319
XR	0.1770	0.1854	1.6118	1.4028	0.3239	0.2329
YC	0.2518	0.2749	1.9846	1.5790	0.3577	0.3423

## Data Availability

The data presented in this study are available on request from the corresponding author.
